# Supplementary material for: Genomic and Biological Characterization of a Novel Proteus mirabilis Phage with Anti-Biofilm Activity
Source: Viruses. 2025 Oct 25;17(11):1419. doi: 10.3390/v17111419 (PMC12656965; doi:10.3390/v17111419)
Supplement: Supplementary file 1 [file viruses-17-01419-s001.zip › viruses-3935040-supplementary.pdf]

Table S1. Host range of phage vB_Pmc_P-07 (P-07)

Strains	Source	Sensitivity	Strains	Source	Sensitivity
P. mirabilis 01	swine	+	*P. mirabilis* 34	swine	-
P. mirabilis 02	swine	-	*P. mirabilis* 35	swine	-
P. mirabilis 03	swine	-	*P. mirabilis* 36	swine	-
P. mirabilis 04	swine	+	*P. mirabilis* 37	swine	-
P. mirabilis 05	swine	-	*P. mirabilis* 38	swine	-
P. mirabilis 06	swine	+	*P. mirabilis* 39	swine	-
P. mirabilis 07	swine	+	*P. mirabilis* 40	swine	-
P. mirabilis 08	swine	+	*P. mirabilis* 41	swine	-
P. mirabilis 09	swine	+	*P. mirabilis* 42	swine	-
P. mirabilis 10	swine	+	*E.coli* 01	swine	-
P. mirabilis 11	swine	-	*E.coli* 02	swine	-
P. mirabilis 12	swine	-	*E.coli* 03	swine	-
P. mirabilis 13	swine	-	*E.coli* 04	swine	-
P. mirabilis 14	swine	-	*E.coli* 05	swine	-
P. mirabilis 15	swine	-	*E.coli* 06	swine	-
P. mirabilis 16	swine	-	*E.coli* 07	swine	-
P. mirabilis 17	swine	-	*E.coli* 08	swine	-
P. mirabilis 18	swine	-	*E.coli* 09	swine	-
P. mirabilis 19	swine	+	*E.coli* 10	swine	-
P. mirabilis 20	swine	+	*E.coli* 11	swine	-
P. mirabilis 21	swine	-	*E.coli* 12	swine	-
P. mirabilis 22	swine	-	*E.coli* 13	swine	-
P. mirabilis 23	swine	-	*E.coli* 14	swine	-
P. mirabilis 24	swine	-	*E.coli* 15	swine	-
P. mirabilis 25	swine	-	*E.coli* 16	swine	-
P. mirabilis 26	swine	-	*E.coli* 17	swine	-
P. mirabilis 27	swine	-	*E.coli* 18	swine	-
P. mirabilis 28	swine	-	*K. Pneumoniae* 01	swine	-
P. mirabilis 29	swine	-	*K. Pneumoniae 02*	swine	-
P. mirabilis 30	swine	-	*K. Pneumoniae 03*	swine	-

P. mirabilis 31	swine	-	*S. aureus*01	cattle	-
P. mirabilis 32	swine	-	*S. aureus*02	cattle	-
P. mirabilis 33	swine	-			

Note: "+": Lytic; "–": Not lytic

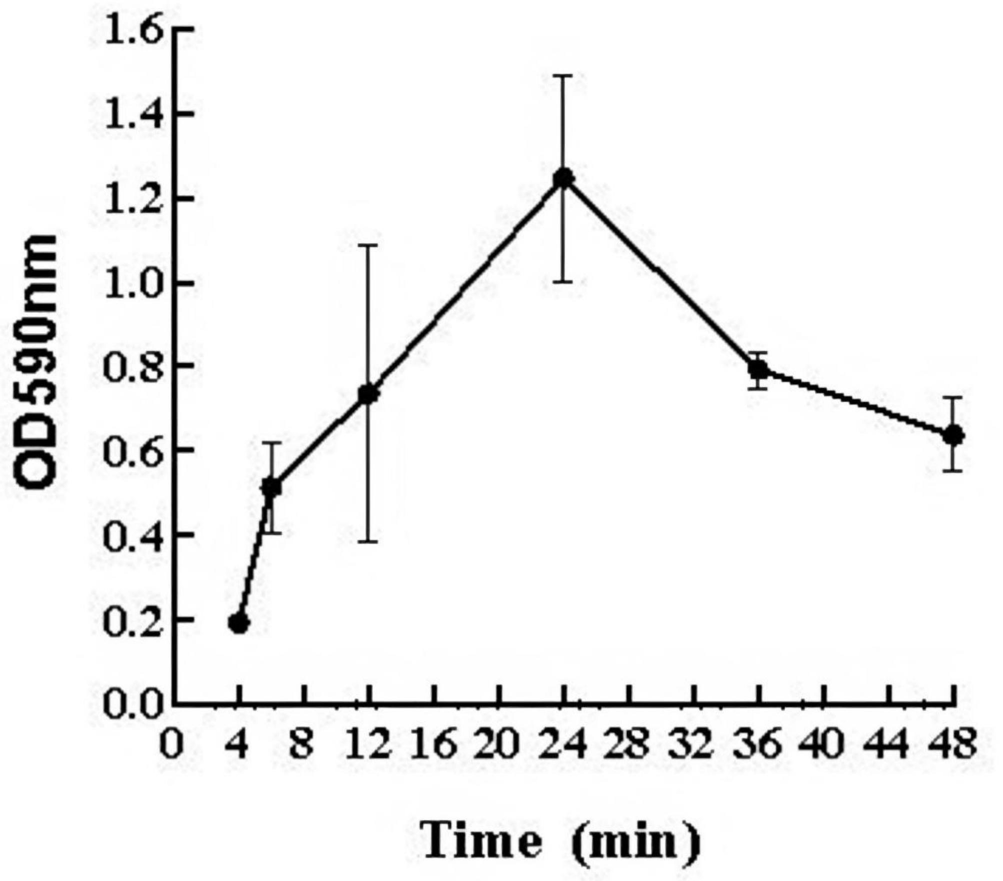

Figure S1. Biofilm formation dynamics of Proteus mirabilis 07. The biofilm biomass was quantified by crystal violet staining at indicated time points (4-48 h). The strain exhibited a typical growth pattern with steady increase from 4 to 24 h, peaking at 24 h, followed by a decline phase.
